# Spatial-temporal changes of iron deposition and iron metabolism after traumatic brain injury in mice

**DOI:** 10.3389/fnmol.2022.949573

**Published:** 2022-08-11

**Authors:** Hao Cheng, Ning Wang, Xingyu Ma, Pengfei Wang, Wenwen Dong, Ziyuan Chen, Mingzhe Wu, Ziwei Wang, Linlin Wang, Dawei Guan, Rui Zhao

**Affiliations:** ^1^Department of Forensic Pathology, School of Forensic Medicine, China Medical University, Shenyang, China; ^2^Liaoning Province Key Laboratory of Forensic Bio-evidence Sciences, Shenyang, China; ^3^Collaborative Laboratory of Intelligentized Forensic Science, Shenyang, China

**Keywords:** traumatic brain injury (TBI), iron deposition, iron metabolism, neuron, oligodendrocyte, astrocyte, microglia

## Abstract

Excessive iron released by hemoglobin and necrotic tissues is the predominant factor that aggravates the outcome of traumatic brain injury (TBI). Regulating the levels of iron and its metabolism is a feasible way to alleviate damage due to TBI. However, the spatial-temporal iron metabolism and iron deposition in neurons and glial cells after TBI remains unclear. In our study, male C57BL/6 mice (8–12 weeks old, weighing 20–26 g) were conducted using controlled cortical impact (CCI) models, combined with treatment of iron chelator deferoxamine (DFO), followed by systematical evaluation on iron deposition, cell-specific expression of iron metabolic proteins and ferroptosis in ipsilateral cortex. Herein, ferroptosis manifest by iron overload and lipid peroxidation was noticed in ipsilateral cortex. Furthermore, iron deposition and cell-specific expression of iron metabolic proteins were observed in the ipsilateral cortical neurons at 1–3 days post-injury. However, iron overload was absent in astrocytes, even though they had intense TBI-induced oxidative stress. In addition, iron accumulation in oligodendrocytes was only observed at 7–14 days post-injury, which was in accordance with the corresponding interval of cellular repair. Microglia play significant roles in iron engulfment and metabolism after TBI, and excessive affects the transformation of M1 and M2 subtypes and activation of microglial cells. Our study revealed that TBI led to ferroptosis in ipsilateral cortex, iron deposition and metabolism exhibited cell-type-specific spatial-temporal changes in neurons and glial cells after TBI. The different effects and dynamic changes in iron deposition and iron metabolism in neurons and glial cells are conducive to providing new insights into the iron-metabolic mechanism and strategies for improving the treatment of TBI.

## Introduction

Traumatic brain injury (TBI), known as the “silent epidemic,” is a sudden insult caused by a mechanical incident. It remains a growing public health concern and is a major contributor to the disability and death of a large number of people worldwide (Dewan et al., 2018). Over 10 million people sustain a TBI each year, causing massive socio-economic losses (Maas et al., 2017). TBIs are composed of primary damage caused by the mechanical impact itself and secondary injury caused by oxidative stress, neuroinflammation, iron overload, and other cytotoxic substances (Pérez de la Ossa et al., 2010; Gu et al., 2011; Hatakeyama et al., 2013; Xie et al., 2014). Once TBI occurs, numerous iron-containing substances are released, disrupting iron homeostasis and exacerbating the impairment of brain trauma. Damaged neural cells are a significant source of free iron, which is a component of numerous proteins, such as ferritin and iron-containing proteases (Zhao and Enns, 2012; Wilkinson and Pantopoulos, 2014). In addition, the mass of free iron, degraded from erythrocytic hemoglobin (Hb) of TBI-induced parenchymal hemorrhage (McGinn and Povlishock, 2015; Salehi et al., 2017), contributes to tissue edema, cytotoxic neuronal damage, and even ferroptosis in injured regions (Pérez de la Ossa et al., 2010; Wang et al., 2016; Zille et al., 2017; Chen Y. et al., 2020; Tang et al., 2020). Many studies have reported that overloaded iron might promote toxic lipid peroxidation and ferroptosis, and further impair neural function in TBI models, numerous neurodegenerative diseases, and intracerebral hemorrhage (Hatakeyama et al., 2013; Li et al., 2016; Xie et al., 2019; Geng et al., 2021). In line with these findings, neuronal impairment could be attenuated by the iron scavenger deferoxamine (DFO) or the ferroptosis inhibitor ferrostatin-1 after brain injury (Hatakeyama et al., 2013; Xie et al., 2019). Therefore, iron homeostasis in the brain is important for the maintenance of physical function.

Physiological conditions, the level of iron is strictly regulated by various iron import, export, and storage proteins. Generally, iron (mainly in the form of ferric Fe^3+^) is imported into the cellular endosome *via* the classic transferrin-transferrin receptor 1 pathway (Muñoz et al., 2011) and transported into the cytoplasm by divalent metal transporter 1 (Fleming et al., 1998). Excessive iron is stored in cytosolic ferritin (composed of ferritin heavy chain [FTH] and ferritin light chain [FTL]) (Theil, 2013) or exported outside the cells through the iron exporter ferroportin1 (FPN1) (Anderson and Vulpe, 2009). An imbalance in the expression of iron metabolism-related proteins may lead to tissue damage. Studies have found that FTH deletion results in oxidative stress (Thompson et al., 2003) and impairment of iron homeostasis in the mouse cortex (Rui et al., 2021). FPN1 deficiency aggravated memory impairment in a rodent Alzheimer’s disease model (Bao et al., 2021). Hence, proteins related to iron metabolism are important for iron homeostasis. Presently, studies have mainly focused on the changes in iron metabolism-related proteins and iron deposition at the tissue level in TBI models (Ward et al., 2014; Rui et al., 2021), there has been no systematic evaluation of the cell type-specific expression of iron metabolism-related proteins and iron deposition.

In this study, dynamic spatial-temporal changes in iron deposition and levels of iron metabolism-related proteins were investigated in mouse controlled cortical impact models. Combined with the iron chelator DFO, the potential effects of intracellular iron overload on different neural cells were explored after TBI.

## Materials and methods

### Animals and ethics statements

Eight-week-old healthy male C57BL/6 mice (*n* = 144, weighing 20–26 g) used in this study were provided by the China Medical University. Mice were housed under a constant temperature (23 ± 1°C), humidity (60%), and a 12-h light-dark cycle, with free access to food and water. The animals were used based on the principle of random assignment. All procedures were performed in accordance with the national guidelines for the care and use of laboratory animals. The protocol for animal experiments was approved by the China Medical University Animal Care and Use Committee (AUP: KT2020135), and the experiments are reported in accordance with ARRIVE guidelines.

### Controlled cortical impact models and sample collection

Controlled cortical impact (CCI) models were conducted as previously described to induce a moderate traumatic brain injury (Dong et al., 2018). Briefly, mice were anesthetized with 4% isoflurane in oxygen and placed on a stereotaxic frame. The mice skull was exposed after making a midline incision, and a portable drill was used to perform a 5-mm craniotomy over the left cerebral hemisphere, which was located 2.5 mm posterior to the bregma and 2.5 mm on the left side of the sagittal suture. The bone flap was removed, and a craniocerebral strike apparatus (PinPoint PCI3000, Hatteras Instruments) was used to cause a vertical impact on the cortex (diameter impactor, 3 mm; velocity, 1.5 m/s; residence time, 50 ms; depth, 1 mm), followed by suturing. Mice in the sham group received the same operation, except for cortical impact.

All mice were divided into the following groups randomly: Sham (sham group, *n* = 18), Sham + Vehicle (Sham-Veh, *n* = 6), Sham + deferoxamine (Sham-DFO, *n* = 6), TBI (TBI group, *n* = 54), TBI + vehicle (Veh group, *n* = 30), and TBI + deferoxamine (DFO group, *n* = 30). The TBI + DFO and TBI + Veh groups were injected intraperitoneally with DFO (50 mg/kg/day, dissolved in saline; MedChemExpress, NJ, United States) or saline, respectively, 15 min after injury, and continued until the day before sample collection. The dose and treatment of DFO were based on those in a previous work (Li et al., 2016). Mice were anesthetized with pentobarbital sodium, followed by heart perfusion with cold phosphate-buffered saline and brain dissection at the indicated times of 1, 3, 7, and 14 days post injury (dpi). For immunohistochemical or immunofluorescence (IF) staining, additional perfusion with 4% paraformaldehyde was performed. The surgical procedures and postoperative care were performed by the same experienced investigator, and investigators were blinded to treatment groups during experimental assays to minimize potential confounders. Benefit from careful and experienced operation, the mice all survived during the process of model conducting, except for some acceptable unavoidable minute differences (such as size of damage area), and a sufficient number of parallel groups ware set up to ensure that the results are believable.

### Perl’s staining

Modified 3,3-diaminobenzidine-Perl’s staining was used to detect iron (mainly ferric Fe^3+^) (Ma et al., 2016). Briefly, paraffin-embedded sections were dewaxed, dehydrated, and then incubated with Perl’s assay (G1422; Solarbio, Beijing, China) for 30 min according to the manufacturer’s instructions. Endogenous peroxidase was scavenged using a 0.3% hydrogen peroxide solution for 10 min. Afterward, the staining signal was developed using DAB for 5 min and counterstained with hematoxylin.

### Terminal deoxynucleotidyl transferase dUTP nick end labeling and Fluoro-Jade C staining

Terminal deoxynucleotidyl transferase dUTP nick end labeling (TUNEL) and Fluoro-Jade C staining were conducted as previous described (Dong et al., 2018). Briefly, TUNEL staining was performed according to the manufacturer’s instructions (A111-01; Vazyme, Nanjing, China). Sections were subjected to deparaffinization, hydrated, treated with equilibration solution, incubated with TUNEL staining assay at 60°C for 1 h, and counterstained with 4t,6-diamidino-2-phenylindole (DAPI). Fluoro-Jade-C (FJC) staining was conducted to detect cell degeneration according to the manufacturer’s instructions (TR-100-FJ; AmyJet Scientific, Wuhan, China).

### Immunofluorescence and co-staining of Perl’s staining with immunofluorescence, terminal deoxynucleotidyl transferase dUTP nick end labeling, or Fluoro-Jade-C staining

Paraffin (4 μm) and frozen (30 μm) sections were used for the IF studies. IF staining was performed as previous described (Ren et al., 2020). Images were acquired using a laser scanning confocal microscope (TCS SP8; Leica). The antibodies used in this study are listed in Supplementary Table 1. Co-staining of Perl’s staining with IF, TUNEL, or FJC staining was conducted as aforementioned protocols and previous described (Ma et al., 2016), of note, the hematoxylin counterstaining were omitted when brain sections were co-stained with specific cellular markers, and the co-staining was conducted initially by Perl’s staining and counterstained by other staining. Notably, the positive iron staining reaction was replaced with pseudowhite for subsequent observation and analysis.

### Protein exaction and Western blotting

Approximately 4 mm of the tissue samples from the cortical area surrounding the ipsilateral-injured cortices (Dong et al., 2019) were collected and homogenized in a RIPA buffer (P0013C; Beyotime Biotechnology, Shanghai, China) containing a protease inhibitor cocktail (P1006; Beyotime Biotechnology, Shanghai, China). A BCA assay kit (P0012; Beyotime Biotechnology, Shanghai, China) was used to quantify the protein concentration. Western blotting (WB) was performed as previously described (Dong et al., 2018). Relative band density was quantified using ImageJ software (National Institutes of Health) and normalized to that of β-ACTIN. Antibody information is presented in Supplementary Table 1.

### Glutathione and malondialdehyde detection

Injured cortical samples were homogenized with 0.9% NaCl and centrifuged to collect the supernatant according to the manufacturer’s instructions. A glutathione (GSH) detection assay (A006-1-1; NanJing JianCheng Bioengineering Institute, Nanjing, China) was used. The malondialdehyde (MDA) levels were measured according to the instructions of the MDA detection assay (A003-1-2; NanJing JianCheng Bioengineering Institute, Nanjing, China). The amount of GSH or MDA was normalized to the total protein level and expressed as GSH/total protein or MDA/total protein.

### Transmission electron microscopy

The mitochondrial ultrastructure in the mouse brain was assessed using transmission electron microscopy. Briefly, ipsilateral-injured cortices were fixed in 2.5% glutaraldehyde for 2 h and 1% osmium tetroxide for 1 h at 4°C, respectively, then dehydrated, and embedded in an epoxy resin. Ultrathin tissue sections were prepared and observed under a transmission electron microscope (CM100; Philips).

### Sholl analysis of the morphology of microglia

Sholl analysis was used to assess the morphology of microglia in the ipsilateral-injured cortex as described by Flygt et al. (2018). Fluorescence images with ionized calcium binding adaptor molecule 1 (IBA1) were converted to an 8-bit format using ImageJ software (National Institutes of Health), and the number of intersections and soma areas was quantified using the ImageJ Sholl analysis plugin. The number of cellular intersections with circles of different diameters indicated the degree of microglial ramification. The soma area was calculated to assess the microglial activation.

### Data collection and statistical analysis

The data are expressed as mean ± standard deviation (SD). The positive cells in ipsilateral injured cortices were counted and analyzed independently by researchers who were not involved in the trial, and middle area of the cortex was observed to ensure the reliability and consistency of the results. Specifically, three sections were taken from each mouse for staining, and three areas of each section were taken for counts of positive cells. The positive cells were counted using ImageJ software, version 6.0 (National Institutes of Health). One-way analysis of variance test and the *post-hoc* Bonferroni test was used to compare data and difference between groups. Student’s *t*-test was used to compare data between the sham and TBI or between the Veh and DFO groups.

GraphPad Prism 8.0 (GraphPad Software) was used to perform the statistical analyses. A *p*-value < 0.05 was considered statistically significant.

## Results

### Abnormal iron deposition and iron metabolism in ipsilateral-injured cortices

**FIGURE 1:**
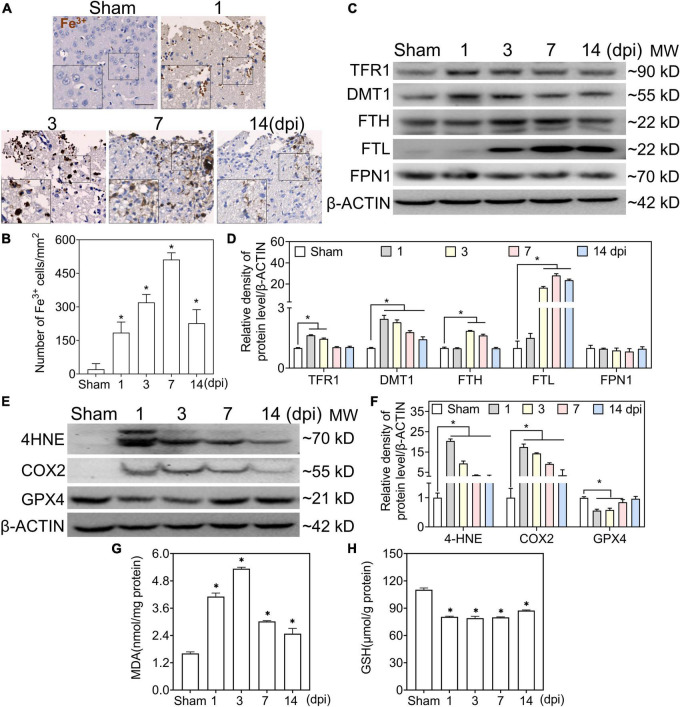
Traumatic brain injury (TBI) accounts for iron accumulation, abnormal iron metabolism, and ferroptosis. **(A)** Representative image of DAB-enhanced Perl’s (Fe^3+^) staining of ipsilateral-injured cortices at the indicated time (scale bar = 50 μm). **(B)** Quantitative analysis of Fe^3+^-positive cells at different intervals after TBI. Data are presented as mean ± SD, and *n* = 6. **(C)** Representative image of Western blotting of TFR1, DMT, FTH, FTL, and FPN1 in injured cortices at the indicated time. **(D)** Relative intensity of proteins in panel **(C)**. Data are presented as mean ± SD, and *n* = 6. **(E,F)** Representative bands and corresponding quantitative analyses of 4-HNE, COX2, and GPX4 in ipsilateral cortices at different time points. Data are presented as mean ± SD, and *n* = 6. **(G,H)** The levels of MDA and GSH in injured cortices at the indicated time. Data are presented as mean ± SD, and *n* = 3. *p < 0.05 versus the sham group. DAB, 3,3-diaminobenzidine; SD, standard deviation; TFR1, transferrin R1; DMT1, divalent metal transporter 1; FTH, ferritin heavy chain; FTL, ferritin light chain; FPN1, ferroportin1; 4-HNE, 4-hydroxynonenal; COX2, cyclooxygenase-2; GPX4, glutathione peroxidase 4; MDA, malondialdehyde; GSH, glutathione.

To test the accumulation of Fe^3+^ in the injured cortical parenchyma, we performed Perl’s staining to detect iron deposits in the ipsilateral cortex. At 1 dpi, a massive iron-positive reaction was localized in clustered erythrocytes-shaped cells and then distributed in neuron-shaped cells at 1–3 dpi, which reached its peak in glia-like cells at 7 dpi (Figures 1A,B). To evaluate the status of iron metabolism after TBI, the levels of iron metabolic proteins were analyzed by Western blotting. As shown in Figures 1C,D, transferrin R1 (TFR1) was slightly increased at 1–3 dpi after injury and returned to normal levels. However, the divalent metal transporter 1 (DMT1) levels continued to increase after injury. Moreover, the FTH level increased gradually between 3 and 7 dpi, and the FTL level increased significantly at 7 dpi. Since intracellular iron overload leads to lipid peroxidation and ferroptosis, the redox state of the injured cortex was evaluated after TBI. As shown in Figures 1E,F, the protein levels of 4-hydroxynonenal (4-HNE) and cyclooxygenase-2 (COX2) increased sharply at 1–3 dpi, and MDA levels showed the same trend (Figure 1G). Glutathione peroxidase 4 (GPX4) and GSH levels decreased intensively (Figures 1E,F,H), indicating that lipid peroxidation and ferroptosis occurred after TBI. To confirm the relationship between iron deposition and neural degeneration, we performed co-staining of FJC and iron; FJC-positive staining showed colocalization with iron-stuffed cells (Supplementary Figures 1A,B). Our data suggest that abnormal iron deposition and metabolism are associated with neural damage in the peripheral area of the injured cortex.

### Neurons were impaired by excessive iron in the early phase of traumatic brain injury

**FIGURE 2:**
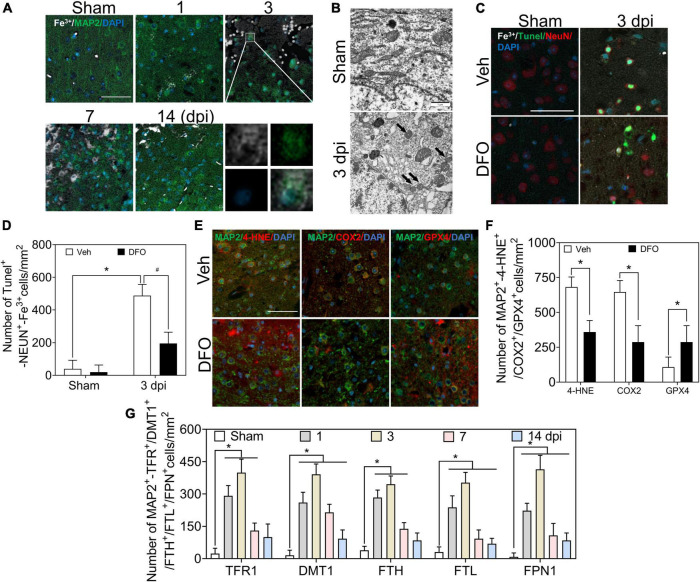
Excessive iron and abnormal iron metabolism impair neurons after TBI. **(A)** Representative co-location image of Fe^3+^ with MAP2 in ipsilateral cortices of mice at the indicated time after TBI (*n* = 6, scale bar = 50 μm). **(B)** Representative transmission electron microscopic images of neuronal mitochondria in ipsilateral cortices of mice in the Sham or TBI group. Shrunken mitochondria with dark density in damaged neurons are indicated by the black arrow at 3 dpi, and *n* = 6. **(C)** Representative colocation image of Fe^3+^, MAP2, and TUNEL in injured cortices of mice after 3 days treatment of Veh or DFO (*n* = 3, scale bar = 50 μm). **(D)** Quantitative analyses of the number of Tunel-NEUN-Fe^3+^-positive cells. Data are presented as mean ± SD. *p < 0.05 versus the Sham group, ^#^*p* < 0.05 versus the Veh group. **(E)** Representative colocation image of 4-HNE, COX2, and GPX4 with MAP2 in injured cortices of mice after 3 days treatment of Veh or DFO (*n* = 3, scale bar = 50 μm). **(F)** Quantitative analysis of the number of 4-HNE/COX2/GPX4-MAP2-positive cells. Data are presented as mean ± SD, *p < 0.05 versus the Veh group. **(G)** Quantitative analysis of the number of TFR1/DMT1/FTH/FTL/FPN1-MAP2-positive cells in an ipsilateral injured cortex at the indicated time. Data are presented as mean ± SD, and *n* = 6. *p < 0.05 versus the Sham group. TBI, traumatic brain injury; MAP2, microtubule associated protein 2; DFO, deferoxamine; Veh, traumatic brain injury + vehicle; dpi, days post-injury; SD, standard deviation; 4-HNE, 4-hydroxynonenal; COX2, cyclooxygenase-2; GPX4, glutathione peroxidase 4; TFR1, transferrin R1; DMT1, divalent metal transporter 1; FTH, ferritin heavy chain; FTL, ferritin light chain; FPN1, ferroportin1.

Co-staining of Perl’s staining with microtubule associated protein 2 (MAP2) IF showed the dynamic accumulation of Fe^3+^ in MAP2-positive cells, and obvious accumulation of iron was detected in neurons at 1–3 dpi (Figures 2A, 6A). To test whether iron accumulation affects neuronal damage, we conducted an experiment on the colocalization of Fe^3+^, MAP2, and TUNEL. Figure 2C shows the iron deposited in the injured neurons. Subsequently, the ultrastructure of the neurons was detected by TEM. As shown in Figure 2B, shrunken mitochondria, characteristic of ferroptotic pathology, were observed in injured neurons. DFO was used as an iron chelator to clarify the roles of iron. Neuronal damage and positive staining of Fe^3+^, MAP2, and TUNEL were ameliorated by DFO treatment in mice with TBI (Figures 2C,D). Next, we explored the mechanism of neuronal death, and the immunoreactions of 4-HNE, COX2, and GPX4 revealed ferroptotic neuronal injuries, which were also mitigated by DFO treatment (Figures 2E,F). Furthermore, the neuronal expressions of TFR1, DMT1, FTH, FTL, and FPN1 were investigated using double IF staining. The number of double-positive cells increased and peaked at 3 dpi (Supplementary Figure 2 and Figure 2G). These observations indicate that abnormal iron metabolism occurs in neurons and that iron contributes to neuronal damage in the early phase of TBI.

### Oxidative stress in astrocytes was iron-independent

**FIGURE 3:**
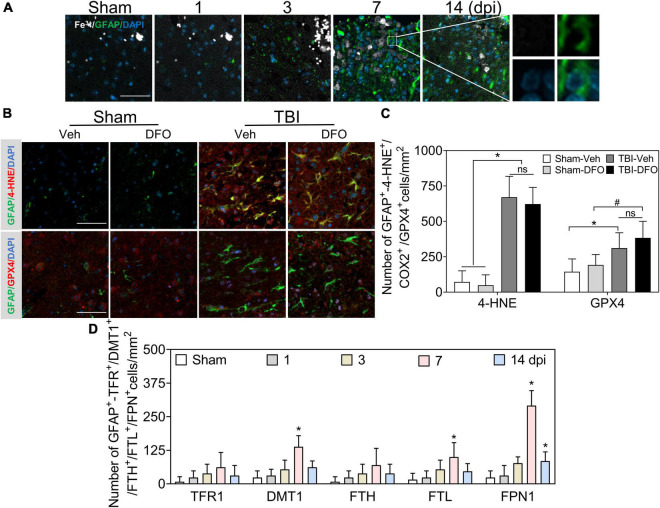
Oxidative stress in astrocytes is irregulated to intracellular iron after TBI. **(A)** Representative colocation image of Fe^3+^ and GFAP in ipsilateral cortices at the indicated time after TBI. (*n* = 6, scale bar = 50 μm). **(B)** Representative colocation image of 4-HNE and GPX4 with GFAP in injured cortices of mice after 7 days treatment of Veh or DFO (*n* = 3, scale bar = 50 μm). **(C)** Quantitative analysis of the number 4-HNE/GPX4-GFAP-positive cells. Interestingly, iron was not detected in astrocytes, while oxidative damage was severe, which could not be ameliorated by DFO. *p < 0.05 versus the Sham-Veh or Sham-DFO group, ^#^*p* < 0.05 vesus the Sham-DFO group. **(D)** Quantitative analysis of the number of TFR1/DMT1/FTH/FTL/FPN1-GFAP-positive cells in injured cortices at the indicated time dpi. Data are presented as mean ± SD, and *n* = 6. *p < 0.05 versus the Sham group. TBI, traumatic brain injury; GFAP, glial fibrillary acidic protein; 4-HNE, 4-hydroxynonenal; GPX4, glutathione peroxidase 4; DFO, deferoxamine; Veh, traumatic brain injury + vehicle; dpi, days post-injury; TFR1, transferrin R1; DMT1, divalent metal transporter 1; FTH, ferritin heavy chain; FTL, ferritin light chain; FPN1, ferroportin1.

**FIGURE 4:**
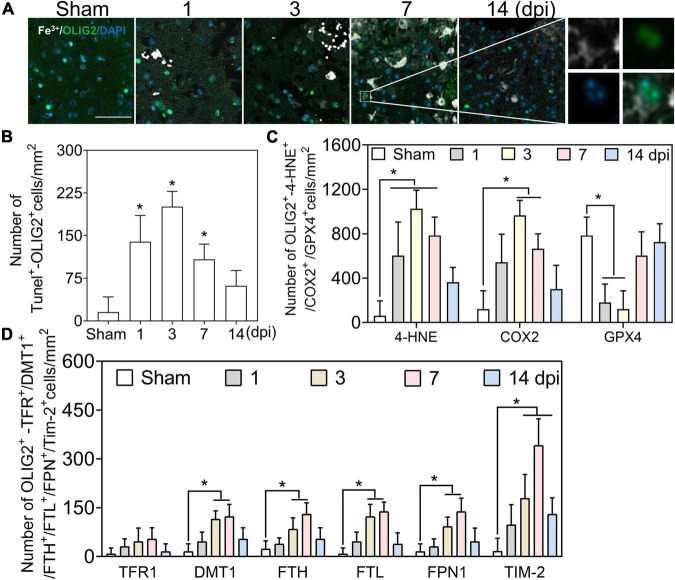
Iron deposited in oligodendrocytes is beyond oxidative stress. **(A)** Representative colocation image of Fe^3+^ and OLIG2 in ipsilateral-injured cortices of mice at the indicated time (*n* = 6, scale bar = 50 μm). The result shows only several Fe^3+^-positive oligodendrocytes at 7–14 dpi. **(B)** Quantitative analysis of the number of positive TUNEL-OLIG2 cells in ipsilateral cortices at the indicated time after TBI. Data are presented as mean ± SD, and *n* = 6. *p < 0.05 versus the Sham group. **(C)** Quantitative analysis of the number of positive 4-HNE/COX2/GPX4-OLIG2 cells in ipsilateral cortices at the indicated time. Data are presented as mean ± SD, and *n* = 6. *p < 0.05 versus the Sham group. **(D)** Quantitative analysis of the number of TFR1/DMT1/FTH/FTL/FPN1/TIM-2-GFAP-positive cells in injured cortices at 3 dpi. Data are presented as mean ± SD, and *n* = 6. *p < 0.05 versus the Sham group. OLIG2, oligodendrocyte transcription factor; dpi, days post-injury; 4-HNE, 4-hydroxynonenal; COX2, cyclooxygenase-2; GPX4, glutathione peroxidase 4; TFR1, transferrin R1; DMT1, divalent metal transporter 1; FTH, ferritin heavy chain; FTL, ferritin light chain; FPN1, ferroportin1.

Reactive astrogliosis occurs after TBI (Zhao et al., 2021). To explore the effect of iron deposition on astrocytic reactions after injury, we conducted co-staining with Fe^3+^ and glial fibrillary acidic protein (GFAP). A large number of GFAP-immunoreactive astrocytes were detected after TBI, while no obvious iron signal was found to colocalize with GFAP (+) cells (Figures 3A, 6A); 4-HNE and GPX4 were strongly expressed in GFAP (+)-reactive astrocytes (Figures 3B,C). To further exclude the possibility that undetectable iron would affect oxidative stress in astrocytes, we used DFO to scavenge free iron. As shown in Figures 3B,C, there was no difference in the immunoreactions of 4-HNE and GPX4 between treatment with or without DFO. We also detected the specific expression of proteins involved in iron metabolism in astrocytes. Only a slight FTL and relatively positive signals of DMT1 and sharply expression of FPN1 were observed in the ipsilateral cortex at 7 dpi (Figure 3D and Supplementary Figure 3), suggesting a strong iron transfer or output ability in astrocytes. Taken together, our data demonstrate that oxidative stress in astrocytes is likely iron-independent, and the high iron transport capability might account for the negative staining of iron in reactive astrocytes.

### Iron deposition in oligodendrocytes was beyond oxidative stress

We first examined the co-staining of iron and the oligodendrocyte marker oligodendrocyte transcription factor (OLIG2). A positive reaction of iron was absent in OLIG2 (+) cells at 1–3 dpi, along with slight deposition in oligodendrocytes at 7 and 14 dpi (Figures 4A, 6A). Positive cell analysis showed that the number of OLIG2 (+) cells decreased in the early phase of TBI and increased from 7 dpi and beyond (Figure 6A). Then, we confirmed oligodendrocyte damage by TUNEL staining after TBI and found that the number of double-positive cells (TUNEL and OLIG2) increased at 1 and 3 dpi (Figure 4B and Supplementary Figure 4A). To examine whether the decline in OLIG2 (+) cells is associated with lipid peroxidation and oxidative stress, we performed immunostaining for 4-HNE, COX2, and GPX4 in oligodendrocytes. As shown in Figure 4C and Supplementary Figure 4B, oxidative stress was observed in the oligodendrocytes. Subsequently, we co-stained iron metabolic proteins (TFR1, DMT1, FTH, FTL, FPN1, and TIM-2) with OLIG2. As shown in Figure 4D and Supplementary Figure 5, the numbers of DMT1, FTH, FTL, and FPN1 in OLIG2 (+) cells increased gradually and reached their maximum at 7 dpi. As expected, the number of TFR1 (+) cells did not differ significantly between the groups at the indicated time points after injury (Figure 4D and Supplementary Figure 5). To further elucidate the alternative iron intake mechanism of oligodendrocytes, we confirmed the upregulated IF signal of TIM-2 (an FTH receptor) in OLIG2 (+) cells (Figure 4D and Supplementary Figure 5). This was consistent with TIM-2 playing a role in iron importation instead of TFR1 in oligodendrocytes (Mukherjee et al., 2020). Our results showed enhanced expression of TIM-2 in oligodendrocytes after injury. In summary, our research demonstrates that iron accumulation in oligodendrocytes plays a role in TBI-induced pathology beyond ferroptosis after TBI.

### Excess iron was related to microgliosis after traumatic brain injury

**FIGURE 5:**
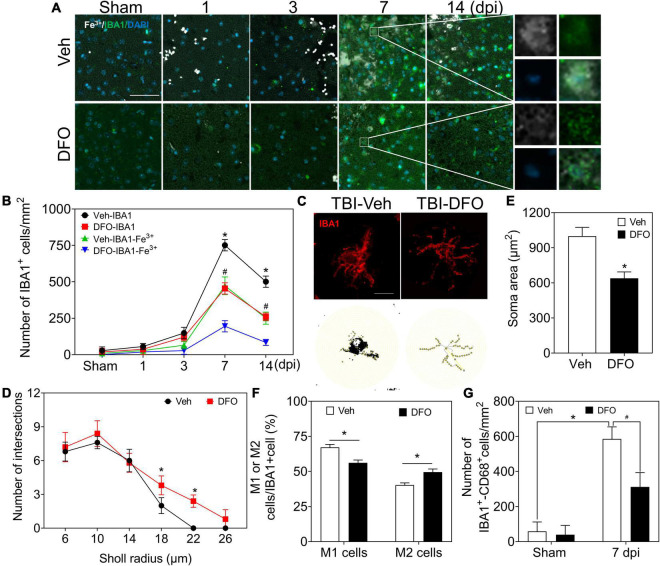
Microglia play a prominent role in iron metabolism, and iron is associated with microgliosis. **(A)** Representative colocation image of Fe^3+^ and IBA1 in ipsilateral-injured cortices of mice treated with Veh or DFO at the indicated time (*n* = 3, scale bar = 50 μm). **(B)** Quantitative analysis of the number of Fe^3+^-IBA1- or IBA1-positive cells in panel **(A)**. Data are presented as mean ± SD. *p < 0.05 versus the Sham group, ^#^*p* < 0.05 versus the DFO group. **(C)** Sholl analysis of the morphology of microglia in injured cortices of mice after 7 days treatment of Veh or DFO (*n* = 6, scale bar = 20 μm). Number of intersections of microglia **(D)** and the soma area of microglia **(E)** in each group. Data are presented as mean ± SD, and *n* = 6. *p < 0.05 versus the Sham group. **(F)** The ratio of M1 cells or M2 cells in injured cortices of mice after 7 days treatment of Veh or DFO. Data are presented as mean ± SD, and *n* = 6, *p < 0.05 versus the Sham group. **(G)** Quantitative analysis of CD68-IBA1-positive cells in injured cortices of mice after 7 days treatment of Veh or DFO, Data are presented as mean ± SD, and *n* = 6. *p < 0.05 versus the Sham group, ^#^*p* < 0.05 versus the Veh group. IBA1, ionized calcium binding adaptor molecule 1; DFO, deferoxamine; Veh, traumatic brain injury + vehicle; SD, standard deviation.

**FIGURE 6:**
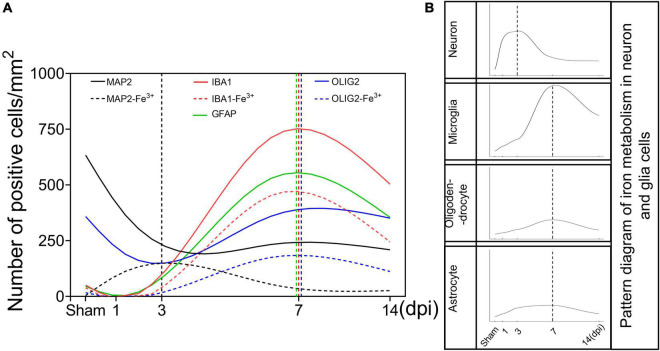
Schematic curves of iron deposition and iron metabolism in neurons and glia cells after TBI. **(A)** Temporal changes of the number of neurons and glia cells and the corresponding number of iron positive cells [black line, MAP2 (+); black dotted line, MAP2 (+)-Fe^3+^ (+); red line, IBA1 (+); red dotted line, IBA1 (+)-Fe^3+^ (+); green line, GFAP (+); blue line, OLIG2 (+); blue dotted line, OLIG2 (+)-Fe^3^^+^ (+)]. **(B)** Temporal change in the pattern diagram of cellular iron metabolism based on the expression of iron metabolism-related proteins in neurons and glial cells. TBI, traumatic brain injury; MAP2, microtubule associated protein 2; IBA1, ionized calcium binding adaptor molecule 1; GFAP, glial fibrillary acidic protein; OLIG2, oligodendrocyte transcription factor.

To explore iron accumulation in microglia, we chose IBA1 as a marker of microglia and conducted experiments on the colocalization of Fe^3+^ and IBA1. Most of the iron-positive signals were overlaid with IBA1 (+) microglia/macrophage cells, which accounted for the type of Fe^3+^ (+) glial-like cells in the late phase of TBI (Figures 5A,B, 6A). Thereafter, microglia-specific expression of iron metabolic proteins was detected, and it was found that TFR1, DMT1, FTH, FTL, and FPN1 were highly expressed in microglia, especially at 7 and 14 dpi (Supplementary Figures 6A,B).

To test the effect of iron accumulation on microgliosis, we calculated the number of IBA1 (+) cells in the TBI groups with or without DFO treatment. The numbers of IBA1 (+) cells and IBA1-Fe^3+^ double-positive cells showed a time-dependent pattern. Both of them decreased sharply after treatment with DFO (Figures 5A,B). We then evaluated microglial morphology after TBI with or without DFO treatment using Sholl analysis. Our results confirmed that the activation of microglia, reflected by the decreased number of branch intersections per radius and the enlarged soma area was ameliorated by DFO administration in mice with TBI (Figures 5C–E). We evaluated the relationship between microglial polarization and iron deposition and found that the ratio of M1 [IBA1 (+)-CD16 (+)] cells was reduced, accompanied by an elevated ratio of M2 [IBA1 (+)-CD206 (+)] cells at 7 dpi after DFO administration (Figure 5F and Supplementary Figure 7A), suggesting that intracellular iron in microglia is associated with microgliosis and their polarization after TBI. To further elucidate the effect of iron deposition on microglial engulfment, CD68 was used to evaluate the phagocytic ability of the microglia. As shown in Figure 6G and Supplementary Figure 7B, DFO alleviated the expression of CD68. In summary, our results demonstrate that iron in microglia is associated with activated microgliosis, their polarization, and engulfment after TBI.

## Discussion

**FIGURE 7:**
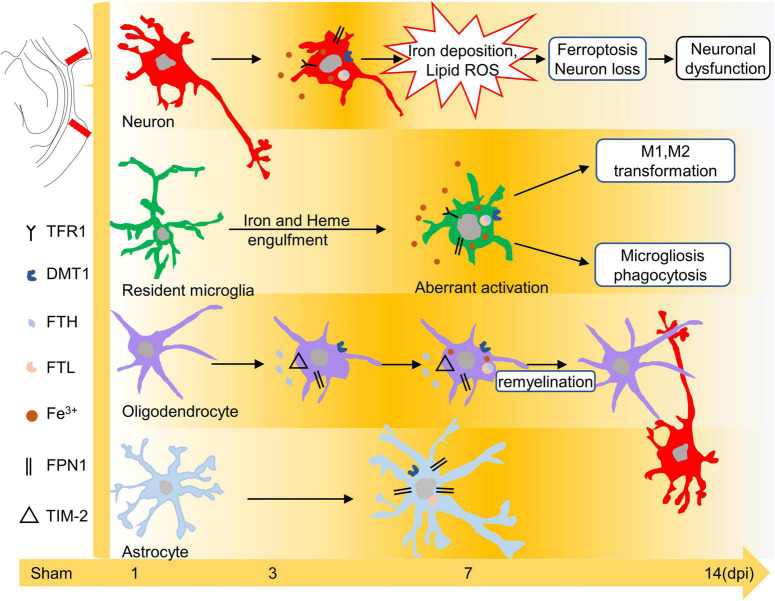
Schematic diagram of the effect of iron or iron metabolism on neurons and glial cells. Iron plays different roles in neurons and glial cells after TBI. Excess iron induces oxidative damage and ferroptosis in neurons during the early phase of TBI. Iron may lead to microglial polarization and microgliosis. In addition, iron overload is likely related to the repair of oligodendrocytes at 7–14 dpi, whereas peroxidative damage in astrocytes is iron-independent. TBI, traumatic brain injury; dpi, days post-injury.

The release of strong iron signals indicates tissues and cell damage after injury, and a large amount of free iron penetrate into interstitial space and cellular, which greatly exacerbate the outcome of TBI. Overloaded iron and ferroptosis play important roles in neuronal impairment and behavior dysfunction after brain trauma. Elucidating spatial-temporal changes in iron and iron metabolism in different cells after TBI is helpful to clarify the mechanism of TBI-induced secondary injuries and provide potential targets for the treatment of TBI. In this study, we explored the changes in iron levels and the dynamic expression of iron metabolic proteins in neurons and glial cells after TBI in mice. The cell type-specific deposition of iron and the distribution of iron metabolism-related proteins are summarized in Figures 6A,B, and the schematic distribution of iron metabolic proteins and the potential roles of iron deposition in neurons and glial cells are presented in Figure 7. Our data contribute to our understanding of the spatial-temporal characteristics and potential functions of iron metabolism in neural cells after TBI, providing new insights into the mechanism underlying iron metabolism in TBI.

Traumatic brain injury leads to primary damage due to the initial impact and secondary injuries caused by delayed neurochemical processes. Several studies have identified different types of regulated cell death, including apoptosis, pyroptosis, necroptosis, parthanatos, and autophagic cell death, after TBI (Lin et al., 2014; Fricker et al., 2018; Yang et al., 2019; Wehn et al., 2021). Recent research has provided evidence that ferroptosis, which is recognized as a new form of iron-dependent programed cell death, contributes to neuronal damage and neural dysfunction in TBI (Kenny et al., 2019) and spinal cord injury (Feng et al., 2021). DFO is an FDA-approved regent to alleviate systemic iron burden in thalassemia major and sickle cell patients (Farr and Xiong, 2021), Its main and most direct role is to chelate and metabolize excess iron out of the body (Elfenbein et al., 2010), thus to play an important cytoprotective role. A large number of experiments also confirmed that DFO can effectively remove iron and play a neuroprotective role (Wu et al., 2011; He et al., 2016; Li et al., 2018). In the present study, lipid peroxidation and neuronal ferroptosis were shown to be highly dependent on iron accumulation after TBI by the use of DFO, which is consistent with findings of previous studies (Hatakeyama et al., 2013). As noted in previous studies, TFR1 participates in the import of iron into cells (Qian and Ke, 2019), and FPN1 is responsible for iron export (Anderson and Vulpe, 2009). DMT1 is responsible for divalent metal ion transportation (Yanatori and Kishi, 2019), ferritin is a complex iron storage protein composed of FTH and FTL (Chen X. et al., 2020) that can store up to 4,500 iron ions (Arosio et al., 2009). In this study, we confirmed that numerous iron metabolic proteins, including TFR1, DMT1, FTH, FTL, and FPN1, were upregulated in neurons in response to TBI, which partly accounts for the complicated mechanism of iron metabolism in neurons after TBI. Interestingly, FPN1 does not appear to change with TBI in the brain tissue lysates compare with the cell-type specific changes in neurons and glia cells, the result of full-size gels also confirmed the credibility of our data of FPN1 (Supplementary Figure 8). In fact, the levels of FPN1 were reported to be regulated by hepcidin, iron levels and the IRP-IRE regulatory system (Bradley et al., 2004; Theurl et al., 2016). Therefore, the characteristics of cell-specific expression changes and multifactorial regulation of FPN1 may be an important reason for the inconsistency between intracellular changes and overall changes, but further research is necessary to disclose the mechanism. Besides, although endogenous protective proteins, such as ferritin and iron exporter FPN1, are upregulated, they are not sufficient to stabilize intracellular iron and prevent TBI-induced ferroptosis in neurons. A recent study reported that conditional knockout of FTH in neurons counteracts the protective effect of melatonin on TBI-induced ferroptosis (Rui et al., 2021), which provides new insight into the prevention or treatment of iron-induced neuronal damage after TBI.

Astrocytes are essential for neural redox homeostasis and for protection against nerve injury (Garg et al., 2011). In our study, robust lipid peroxidation was observed in astrocytes after TBI, which may relate to the imbalanced redox in the interstitial microenvironment of the injured cortex (Qian et al., 2021; Han et al., 2022). In addition, previous studies have shown that astrocytes do not have a high metabolic requirement for iron (Cheli et al., 2020). In the present study, iron deposition was not detected in astrocytes, even in reactive and scar-formed astrocytes, which may be attributed to the special distribution of iron-related metabolic proteins in astrocytes after TBI. The absence of TFR1 immunoreactivity in cortical astrocytes after TBI restricted iron importation, which is consistent with previously reported data (Moos, 1996; Moos et al., 2007). Evidence supports the opinion that astrocytes are inclined to transport iron to other cells rather than to store it (Dringen et al., 2007; Biasiotto et al., 2016). Thus, they provide little iron storage capacity and possess a strong ability for iron transportation. This viewpoint is also supported by our present data showing slight immunoreaction of FTL and upregulated levels of FPN1 and DMT1 in astrocytes after TBI. The characteristics of the iron metabolism in astrocytes after TBI require further investigation.

Oligodendrocytes are the iron-rich cells in the brain with strong iron requirements and metabolic capacity (Reinert et al., 2019; Cheli et al., 2020). After TBI, intracellular iron deposition in OLIG2 (+) cells was only observed at 7 and 14 dpi, which lagged behind cell damage and lipid peroxidation, suggesting that the intricate role of iron in oligodendrocytes is beyond oxidative stress. A previous study (Todorich et al., 2011) and our present results showed that TFR1 was undetectable in mature oligodendrocytes. Instead of the absence of TFR1, an alternative pathway of iron intake in oligodendrocytes is TIM-2 (receptor of FTH), as reported by Todorich et al. (2008). Our results of TIM-2 immunostaining in oligodendrocytes accounted for iron intake and iron deposition in oligodendrocytes at 7–14 dpi. Previous studies have shown that iron is required for oligodendrocyte precursor cell proliferation and differentiation (Lee et al., 2019). Iron is also necessary for oligodendrocyte myelination and remyelination following injury (Todorich et al., 2009; Lee et al., 2019). In our study, dynamic changes in the levels of DMT1, FTH, and FTL might contribute to the healing process of oligodendrocytes after TBI. Moreover, FTH is recognized as an antioxidant iron storage protein (Chen X. et al., 2020) that has a protective effect on oligodendrocytes after TBI (Mukherjee et al., 2020). However, the role of iron in promoting oligodendrocyte turnover requires further exploration.

Studies have found that both blood-derived macrophages and resident microglia migrate to injured regions, which are involved in neuroinflammation and cellular debris engulfment after TBI (Hanisch and Kettenmann, 2007; Herzog et al., 2019). Many studies have provided evidence that microglia/macrophages are also closely related to iron clearance and metabolism in rodent brain ischemia models (Zhao et al., 2007; Shin et al., 2018). The heterogeneity of microglia/macrophages is highly dependent on the origin of cells after injuries (Kroner et al., 2014; Jassam et al., 2017). We confirmed that iron-enriched cells at 7 dpi were almost entirely labeled with TMEM119, a marker for resident microglia (Zrzavy et al., 2018) (data not shown), demonstrating that resident microglia rather than blood-derived macrophages are the main factors playing a role in iron metabolism. Through phagocytosing Hb and necrotic cortical tissues, microglia play a crucial role in iron elimination from the interstitial space or cells, preventing secondary injuries caused by iron overloaded (Morris et al., 1992; Andersen et al., 2014). In addition, microglia exert a significant effect on iron storage by enhancing the expression of ferritin (Zhang et al., 2006; Ashraf et al., 2018), which in turn reduces the level of intracellular free iron. In our study, microglia exhibited a strong capacity for iron intake and storage, manifested as sharp expressions of TFR1, DMT1, FTH, and FTL, indicating the predominant role of microglia in iron metabolism. Microglia are highly malleable and may transform into different subtypes, depending on the environment (Kroner et al., 2014; Jassam et al., 2017). Studies have confirmed that iron affects the physiological characteristics of microglia (Zhang et al., 2017) and their polarization (Kroner et al., 2014; van Vliet et al., 2020), including the enhanced ability of engulfment and the transformation of microglia from M2 to M1 subtypes. Our results demonstrated that microglia with accumulated iron tended to undergo M1 transformation, upregulated phagocytosis, and ameboid-like morphological changes. The relationship between iron deposition and microglial activation was also supported by evidence that microgliosis was eliminated by DFO administration. Microglia, the double-edged sword in the healing process of TBI, play both protective and destructive roles and result in different outcomes of injury (Wang et al., 2013; Kumar et al., 2016). Considering that regulating iron metabolism is beneficial for microglia polarization (Kroner et al., 2014; Yauger et al., 2019), iron metabolism in microglia may provide an efficient target for the treatment of TBI.

In the present study, we illustrated the spatial-temporal changes in iron and iron metabolism-related proteins in neurons and glial cells, as well as the potential role of iron after TBI. However, there are still some limitations. First, we did not explain the underlying cell-specific molecular mechanisms that trigger the transportation and metabolism of iron, and future work is needed to explore the effects of overloaded iron on the function of different neural cells except ferroptosis.

In conclusion, iron metabolism and iron deposition are spatial-temporal changes following TBI. Iron overload and abnormal expression of iron metabolism-related proteins lead to neuronal damage during the early stages of TBI. Oxidative stress in astrocytes after brain contusion is iron-independent. Iron may be associated with the turnover of oligodendrocytes in the late stages of TBI. Overloaded iron is associated with the activation and polarization of microglia. Elucidating the spatial-temporal changes in iron deposition and iron metabolism-related proteins would provide new insights into the mechanisms underlying iron metabolism in TBI, which are beneficial for the treatment of TBI.

## Data availability statement

The original contributions presented in this study are included in the article/Supplementary material, further inquiries can be directed to the corresponding author.

## Ethics statement

This animal study was reviewed and approved by the China Medical University Animal Care and Use Committee (AUP: KT2020135).

## Author contributions

HC, NW, and XM performed the research, analyzed the data, and wrote the manuscript. WD, ZC, MW, ZW, PW, and LW were involved in the experiments and data analyses. RZ and DG designed the study and edited the manuscript. All authors have read and approved the final manuscript.

## Abbreviations

TBI: traumatic brain injury
Hb: hemoglobin
FTH: ferritin heavy chain
FTL: ferritin light chain
FPN1: ferroportin1
IF: immunofluorescence
DAB: 3,3-diaminobenzidine
MDA: malondialdehyde
IBA1: ionized calcium binding adaptor molecule 1
TFR1: transferrin R1
DMT1: divalent metal transporter 1
4-HNE: 4-hydroxynonenal
MAP2: microtubule associated protein 2
GFAP: glial fibrillary acidic protein
OLIG2: oligodendrocyte transcription factor.
