# Supplementary material for: Spatial-temporal changes of iron deposition and iron metabolism after traumatic brain injury in mice
Source: Front Mol Neurosci. 2022 Aug 11;15:949573. doi: 10.3389/fnmol.2022.949573 (PMC9405185; doi:10.3389/fnmol.2022.949573)
Supplement: Supplementary file 1 [file Data_Sheet_1.pdf]

Supplementary Material

Supplementary Table 1. Antibody information used in this study

Antibody	Host	Distributor	Cat.No.	Dilution
**Primary antibody**				
MAP2	Rabbit	Proteintech	17490-1-AP	1:400 (IF)
OLIG2	Goat	R&D Systems	AF2418	1:400 (IF)
GFAP	Rabbit	Proteintech	16825-1-AP	1:400 (IF)
IBA1	Goat	Abcam	Ab5076	1:400 (IF)
FTH	Mouse	Santa Cruz Biotechnology	sc-376574	1:100 (IF)<br>1:500 (WB)
FTL	Mouse	Santa Cruz Biotechnology	sc-390558	1:100 (IF)<br>1:500 (WB)
4-HNE	Mouse	R&D Systems	MAB3249	1:400 (IF)<br>1:1000 (WB)
GPX4	Mouse	Santa Cruz Biotechnology	sc-166570	1:100 (IF)<br>1:500 (WB)
TFR1	Mouse	Thermo Scientific	13-6800	1;400 (IF)<br>1:1000 (WB)
DMT1	Mouse	Santa Cruz Biotechnology	sc-166884	1:100 (IF)<br>1:500 (WB)
FPN1	Mouse	Novus Biology	NBP1-21502	1:400 (IF)<br>1:1000 (WB)
COX2	Mouse	Proteintech	66351-1-Ig	1:400 (IF)<br>1:1000 (WB)
β-ACTIN	Mouse	Proteintech	23660-1-AP	1:1000 (WB)
**Secondary antibody**				
Donkey anti-mouse IgG, Alexa Fluor 488	Donkey	Thermo Scientific	A-21202	1:400 (IF)
Donkey anti-mouse IgG, Alexa Fluor 594	Donkey	Thermo Scientific	A-21203	1:400 (IF)
Donkey anti-rabbit	Donkey	Thermo Scientific	A-21206	1:400 (IF)

IgG, Alexa Fluor 488				
Donkey anti-rabbit IgG, Alexa Fluor 594	Donkey	Thermo Scientific	A-21207	1:400 (IF)
Goat anti-mouse IgG	Goat	Thermo Scientific	ZB-2305	1:1000(WB)
Goat anti-rabbit IgG	Goat	Thermo Scientific	ZB-2301	1:1000(WB)

MAP2, microtubule associated protein 2; OLIG2, oligodendrocyte transcription factor; GFAP, glial fibrillary acidic protein; IBA1, ionized calcium binding adaptor molecule 1; 4-HNE, 4-hydroxynonenal; GPX4, glutathione peroxidase 4; TFR1, transferrin R1; DMT1, divalent metal transporter 1; FPN1, ferroportin 1; COX2, cyclooxygenase-2; Ig, immunoglobulin; IF, immunofluorescence; WB, Western blotting.

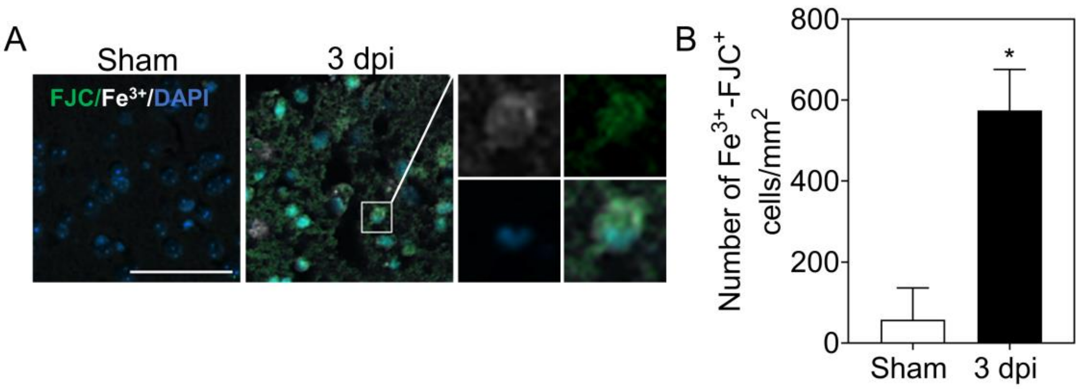

Supplementary Figure 1. Iron-deposited cells are FJC-positive stained after TBI

(A) Representative co-stained image of Perl's staining and FJC staining in injured cortices at 3 dpi (n = 6, scale bar = 50 μm). (B) Quantitative analysis of the number of Fe^{3+}-FJC-positive cells. Data are presented as mean ± SD. * $p < 0.05$ versus the Sham group. TBI, traumatic brain injury; FJC, Fluoro-Jade-C; dpi, days post-injury; SD, standard deviation.

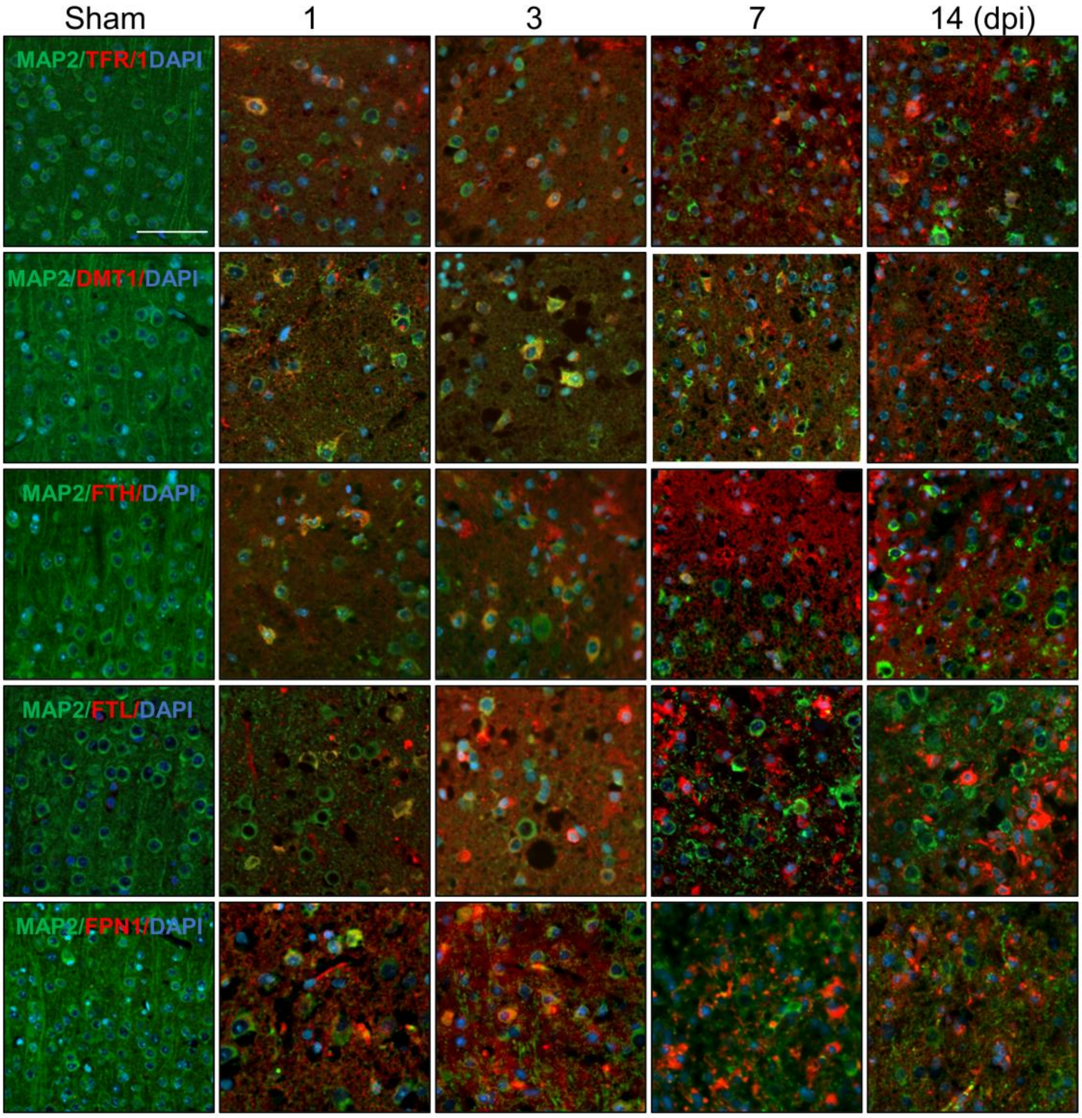

Supplementary Figure 2. Expression of iron metabolism-related proteins in neurons in ipsilateral injured cortices

Representative colocalization image of Fe^{3+} and MAP2 in injured cortices at the indicated time (n = 6, scale bar = 50 μm). TFR1, transferrin R1; DMT1, divalent metal transporter 1; FTH, ferritin heavy chain; FTL, ferritin light chain; FPN1, ferroportin 1; MAP2, microtubule associated protein 2.

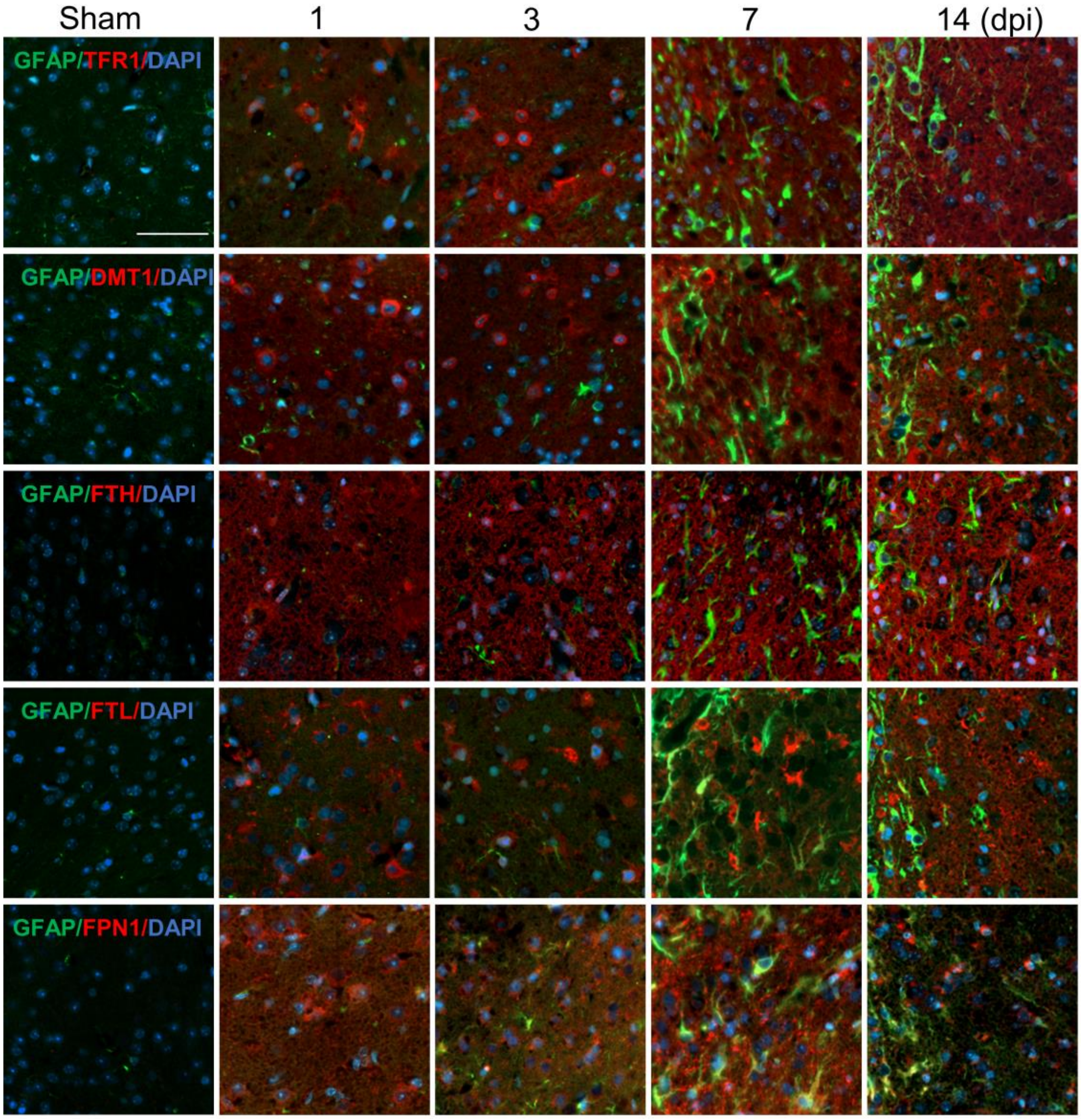

Supplementary Figure 3. Expression of iron metabolic proteins in astrocytes in ipsilateral injured cortices

Representative colocalization image of TFR, DMT1, FTH, FTL, and FPN1 with GFAP in ipsilateral injured cortices at the indicated time (n = 6, scale bar = 50 μm). TFR1, transferrin R1; DMT1, divalent metal transporter 1; FTH, ferritin heavy chain; FTL, ferritin light chain; FPN1, ferroportin 1; GFAP, glial fibrillary acidic protein.

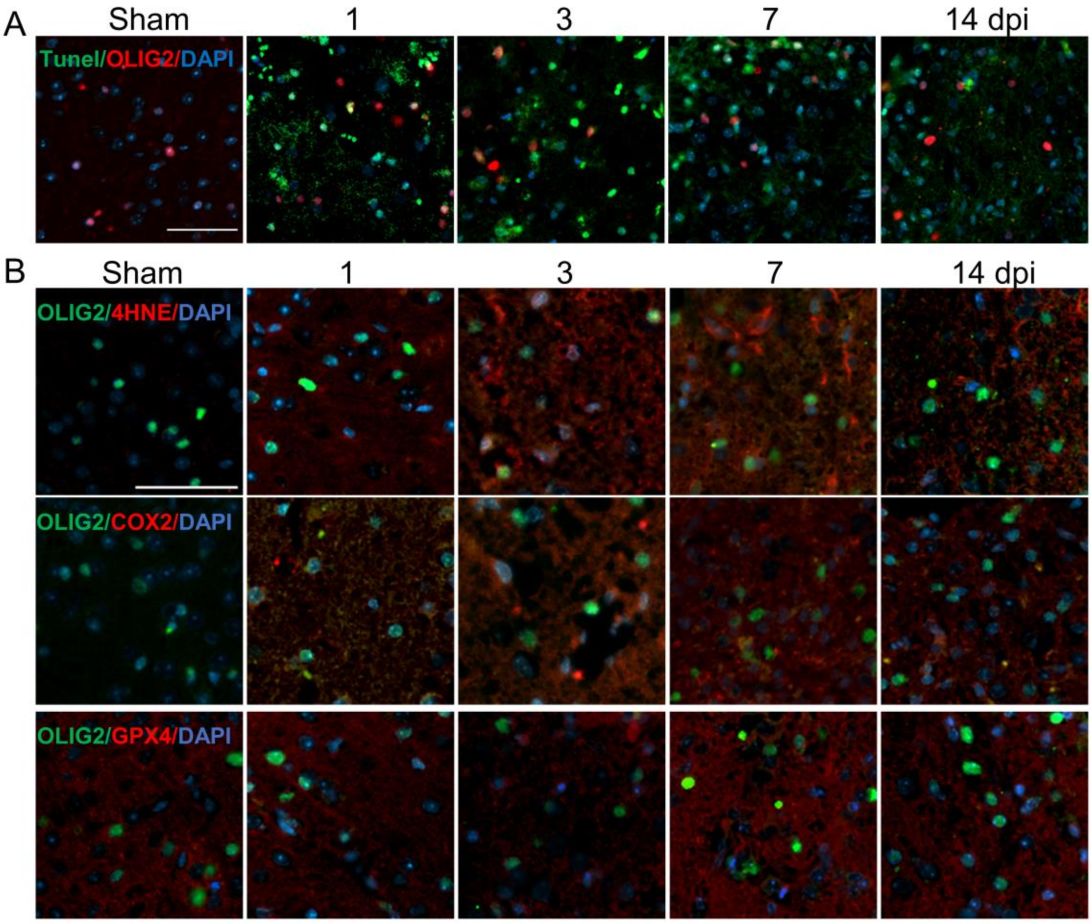

Supplementary Figure 4. TBI leads to impairment of oligodendrocytes

(A) Representative colocation image of TUNEL with OLIG2 in ipsilateral injured cortices at the indicated time (n = 6, scale bar = 50 μm). (B) Representative colocation image of 4-HNE, COX2, and GPX4 with OLIG2 in injured cortices (n = 6, scale bar = 50 μm). TBI, traumatic brain injury; 4-HNE, 4-hydroxynonenal; COX2, cyclooxygenase-2; GPX4, glutathione peroxidase 4; OLIG2, oligodendrocyte transcription factor.

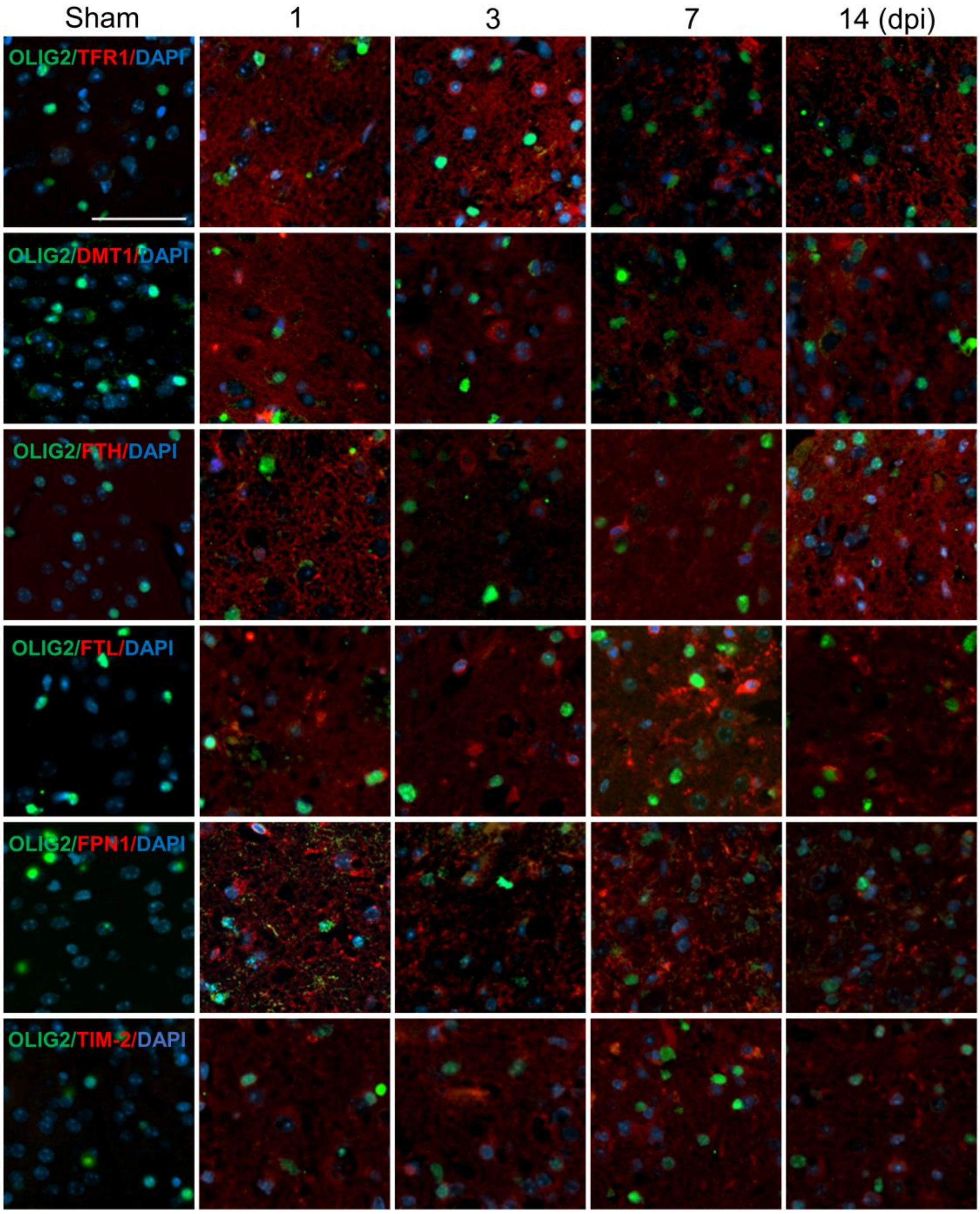

Supplementary Figure 5. Expression of iron metabolic proteins in oligodendrocytes in ipsilateral injured cortices

Representative colocation image of TFR, DMT1, FTH, FTL, FPN, and TIM-2 with OLIG2 in ipsilateral injured cortices at the indicated time (n = 6, scale bar = 50 μm). TFR1, transferrin R1; DMT1, divalent metal transporter 1; FTH, ferritin heavy chain;

FTL, ferritin light chain; FPN1, ferroportin 1; OLIG2, oligodendrocyte transcription factor.

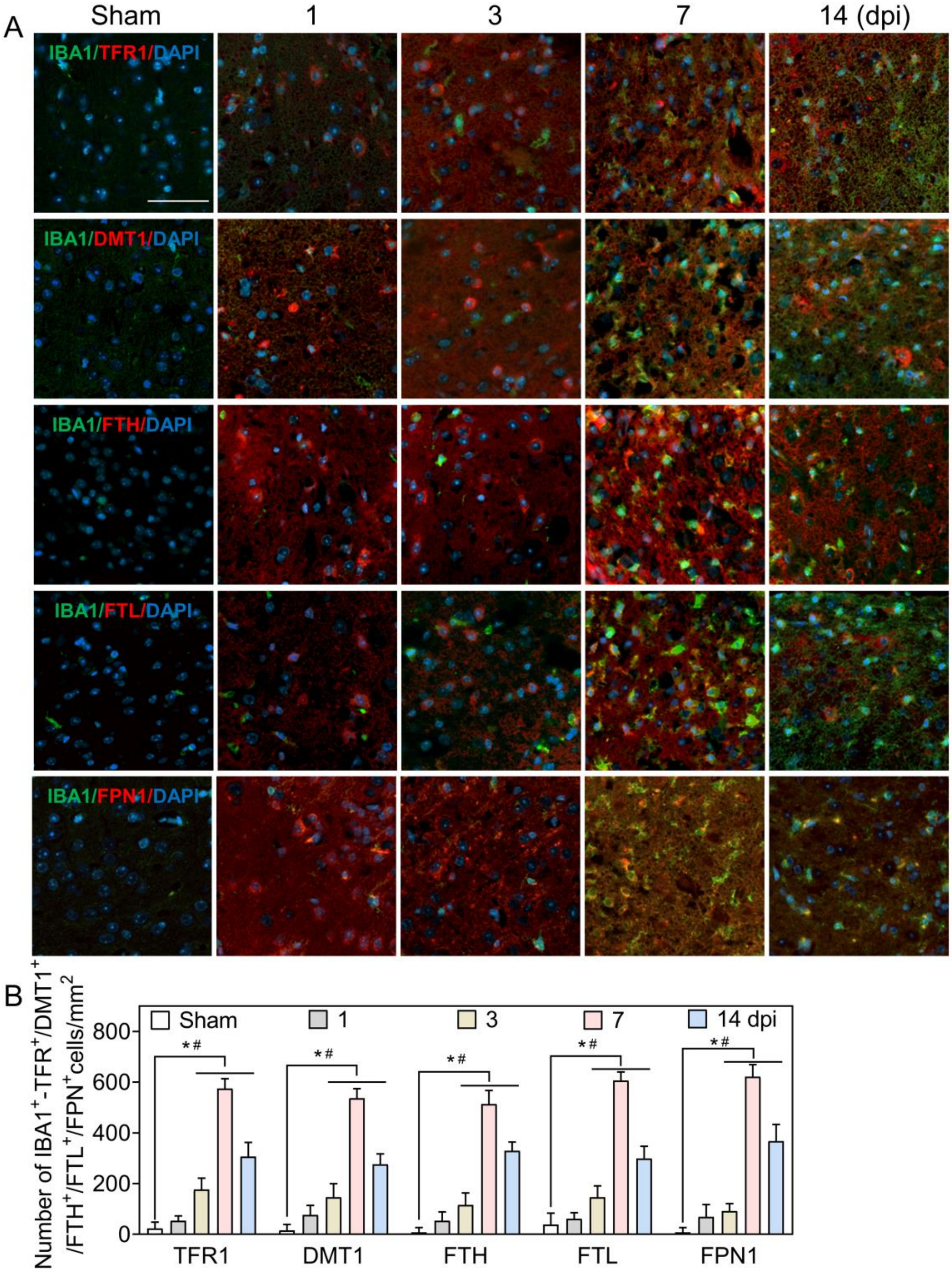

Supplementary Figure 6: Expression of iron metabolic proteins in microglia in

ipsilateral injured cortices

(A) Representative colocation image of TFR, DMT1, FTH, FTL, and FPN with IBA1 in injured cortices at the indicated time (n = 6, scale bar = 50 μm). (B) Quantitative analysis of the number of TFR/DMT1/FTH/FTL/FPN-IBA1-positive cells. Data are presented as mean ± SD, and n = 6. * $p < 0.05$ versus the Sham group, # $p < 0.05$ versus the former group. TFR, transferrin R; DMT1, divalent metal transporter 1; FTH, ferritin heavy chain; FTL, ferritin light chain; FPN1, ferroportin 1; IBA1, ionized calcium binding adaptor molecule 1.

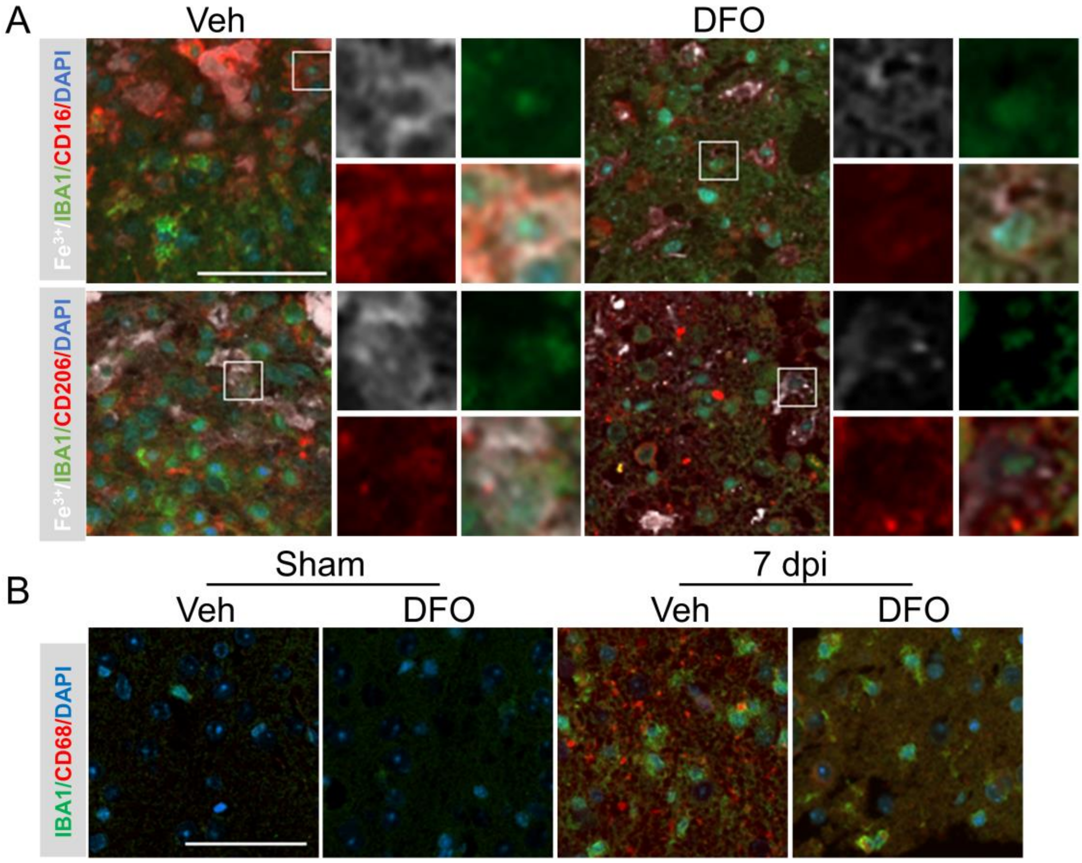

Supplementary Figure 7: The polarization of microglia and its expression of CD68 after DFO treatment

(A) Representative colocation image of Fe^{3+} with IBA1-CD16 or IBA1-CD206 in ipsilateral cortices of mice in TBI groups after 7days treatment of Veh or DFO (n = 6, scale bar = 50 μm). (B) Representative colocation image of CD68 with IBA1 in injured cortices at 7 dpi after treatment with Veh or DFO (n = 6, scale bar = 50 μm). IBA1, ionized calcium binding adaptor molecule 1; Veh, traumatic brain injury+vehicle; dpi, days post-injury.

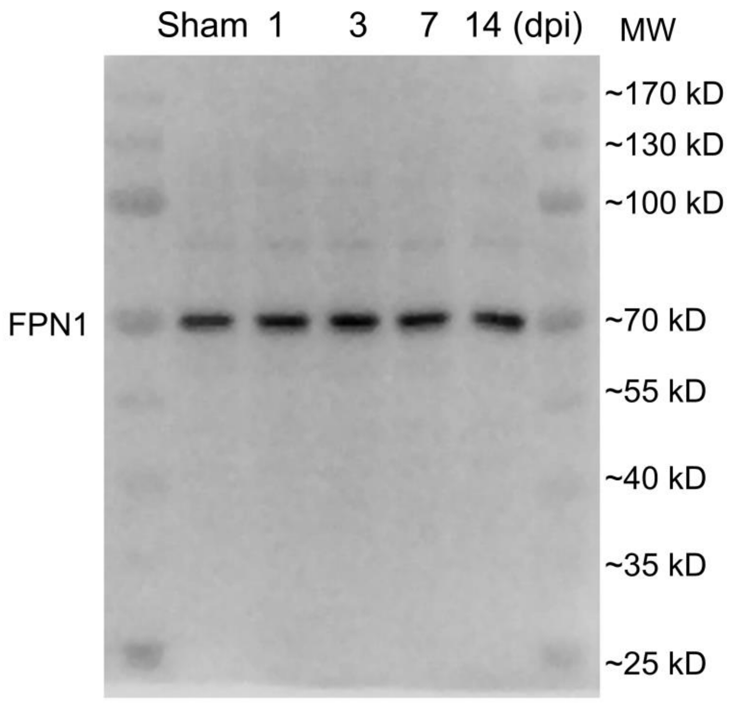

Supplementary Figure 8：the PVDF membrane image of a Full-sized gels of FPN
